# Supplementary material for: Coupling chemical mutagenesis to next generation sequencing for the identification of drug resistance mutations in Leishmania
Source: Nat Commun. 2019 Dec 9;10:5627. doi: 10.1038/s41467-019-13344-6 (PMC6901541; doi:10.1038/s41467-019-13344-6)
Supplement: Supplementary file 1 — Supplementary Information [file 41467_2019_13344_MOESM1_ESM.pdf]

Supplementary Figures and Tables to:

Coupling Chemical Mutagenesis to Next Generation Sequencing for the identification of drug resistance mutations

Bhattacharya, A., et al.

Supplementary Figures

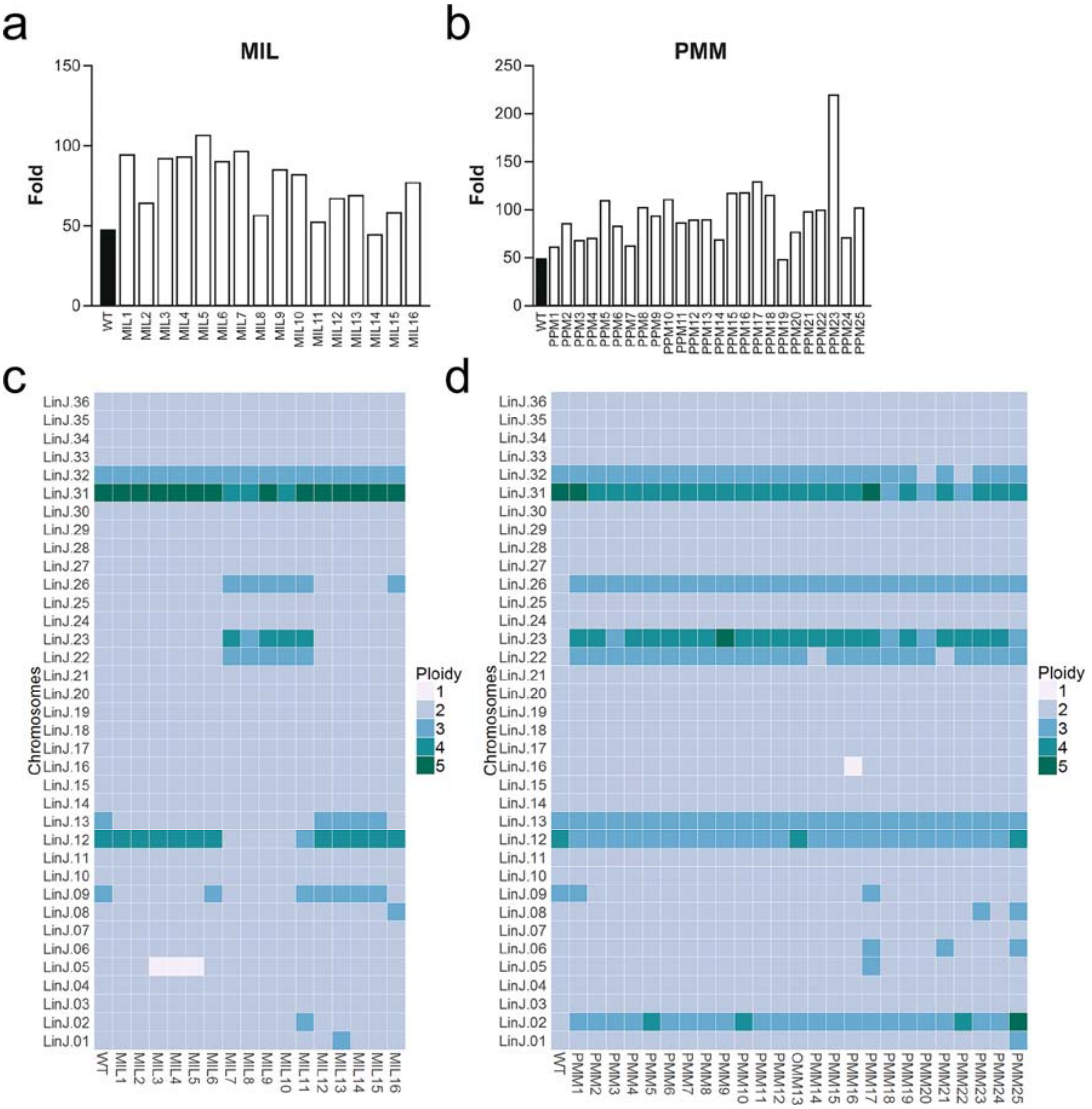

Supplementary Figure 1. **Genomic coverage and changes in ploidy in drug resistant *Leishmania*.** Average fold genomic coverage for wild-type and mutants resistant to MIL (**a**) and PMM (**b**) is plotted. Chromosome ploidy in wild-type and mutants resistant to MIL (**c**) and PMM (**d**). We calculated the ratio between median FPKM values for individual chromosomes and whole genome for each strain. Source data are provided as a Source Data file.

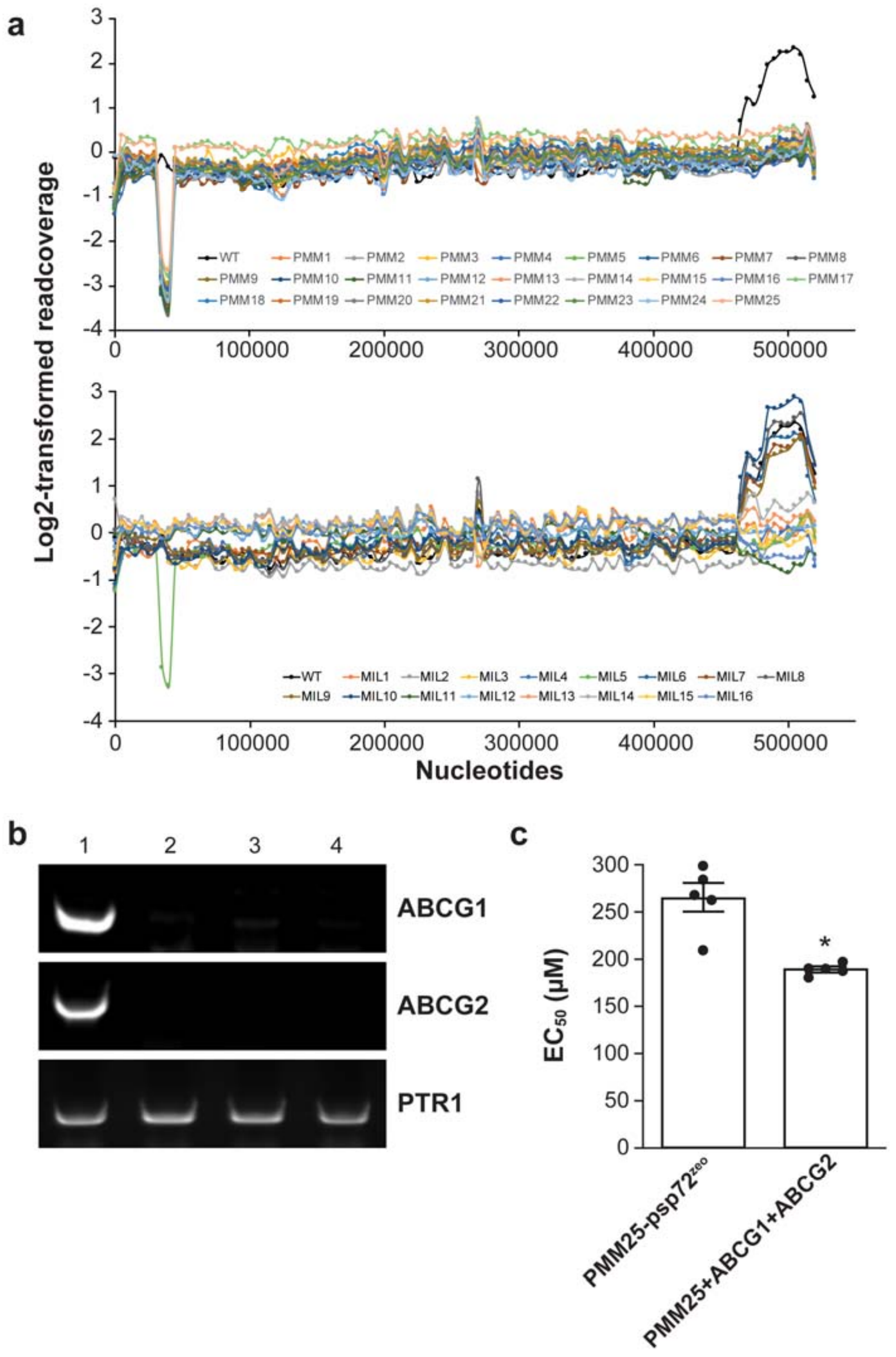

Supplementary Figure 2. **Deletion of a specific locus in chromosome 6 of paromomycin resistant *Leishmania*. a,** CNV for chromosome 6 as derived from read depth coverage of uniquely mapped reads along small non-overlapping genomic windows (5 kb) for PMM mutants (top) and MIL mutants (bottom). Read counts were log2-

transformed and normalized to the total number of uniquely-mapped reads for each strain. A deletion of 35-40 kb was predicted in every PMM-resistant mutants (and in the MIL5 mutant), but not for the wild-type clone (black circles) used for initiating chemical mutagenesis. **b,** The deletion was confirmed by PCR amplification of *ABCG1* and *ABCG2*, two genes encoded in the deleted locus. Wild-type (1), PMM5 (2), PMM16 (3) and PMM25 (4). The *PTR1* gene was amplified as a positive control. **c,** The genes *ABCG1* and *ABCG2* were episomally expressed from the psp72αZEOα plasmid in PMM25 and found to confer a modest sensitization to paromomycin in that mutant. The mutant was also transfected with empty psp72αZEOα (PMM25-psp72$^{\alpha ZEO\alpha}$) as a control. Data are mean ± SEM for n=5 independent biological replicates. Statistical analyses were performed using unpaired two-tailed t-tests. *P < 0.05. Source data are provided as a Source Data file.

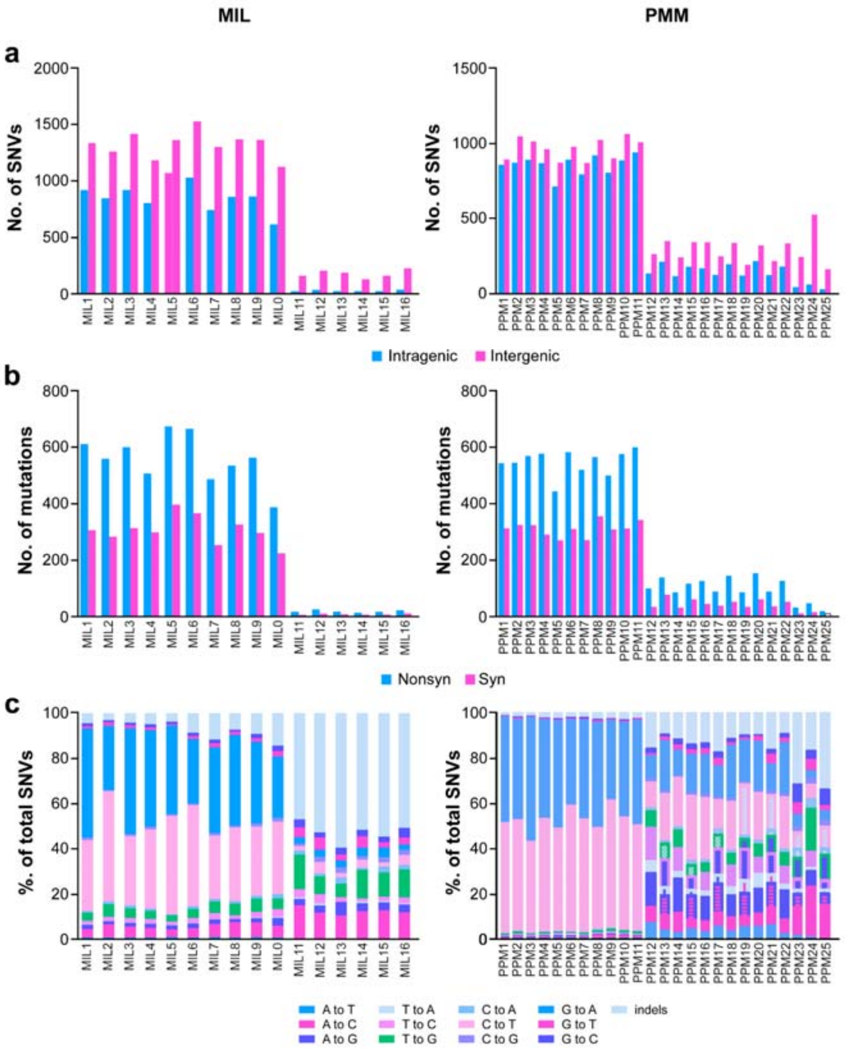

Supplementary Figure 3. **Mutation type in drug resistant *Leishmania* following chemical mutagenesis. a,** Number of intragenic and intergenic SNVs detected in *L. infantum* mutants resistant to MIL (left panel) and PMM (right panel) compared to the wild-type parent clone. **b,** Number of synonymous and non-synonymous mutations detected for intragenic SNVs across mutants selected against MIL (left panel) and PMM (right panel). Bar colours represents the type of mutations detected. **c,** Depiction of base specific variation profile for SNVs in each individual mutant clones. Bar colours reflect the type of variation determined. Source data are provided as a Source Data file.

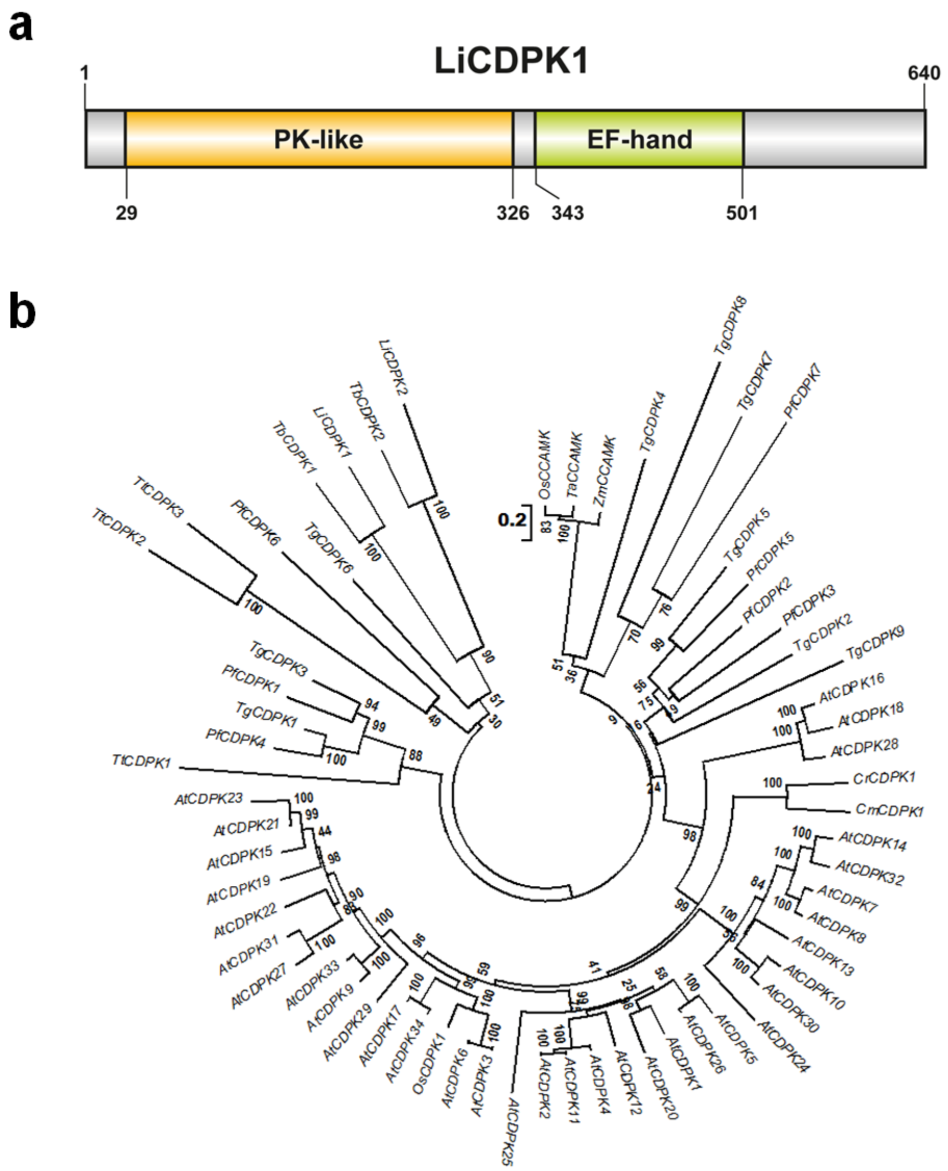

Supplementary Figure 4. **CDPK1 and CDPK1 mutations. a,** CDPK1 is presented with its protein kinase (PK-like) and Ca^{2+}-binding (EF-hand) domains. The PK-like domain was determined to resemble AMPK/CaMK superfamily by conserved domain (CD)-search (NCBI). **b,** Phylogenetic analysis of direct calcium binding kinases (CDPK and CCAMK). Sixty-three sequences of the kinase category orthologues comprising representatives of various domains of life were analyzed by CLUSTALΩ. A Newik phylogenetic tree was constructed for the alignment with *MEGA6* by Neighbour-Joining method with 10000

bootstraps, computed by JTT analysis. Bootstrap values are depicted at each branch node depicting the evolutionary proximity of the proteins. OS: *Oryza sativa* (OsCCAMK: Q6AVM3.1 and OsCDPK1: XP_015650814.1); Ta: *Triticumaestivum* (TaCCAMK: ADK22086.1); Zm: *Zia maize* (ZmCCAMK: NP_001105906.1); Cr: *Chlamydomonasreinhardtii* (CrCDPK1: XP_001691570.1); Cm: *Chlamydomonas moewusii* (CmCDPK1: CAA89202.1); At: *Arabidopsis thaliana* (AtCDPK1: NP_196107.1; AtCDPK2: NP_174807.1; AtCDPK3: Q42479.1; AtCDPK4: NP_192695.1; AtCDPK5: AEE86498.1; AtCDPK6: NP_194096.1; AtCDPK7: NP_568281.1; AtCDPK8: Q42438.1; AtCDPK9: Q38868.1; AtCDPK10: Q9M9V8.1; AtCDPK11: Q39016.2; AtCDPK12: Q42396.1; AtCDPK13: AEE78852.1; AtCDPK14: NP_181717.3; AtCDPK15: NP_001190794.1; AtCDPK16: NP_179379.1; AtCDPK17: NP_196779.1; AtCDPK18: NP_001190932.1; AtCDPK19: NP_176386.2; AtCDPK20: NP_181425.1; AtCDPK21: AEE82416.1; AtCDPK22: Q9ZSA3.2; AtCDPK23: AEE82419.1; AtCDPK23: NP_001190672.1; AtCDPK24 NP_180708.1; AtCDPK25: AEC09174.1; AtCDPK26: NP_001190950.1; AtCDPK27: NP_192379.2; AtCDPK28: NP_851280.1; AtCDPK29: NP_974150.2; AtCDPK30: NP_177612.2; AtCDPK31: NP_680596.2; AtCDPK32: NP_191312.2; AtCDPK33: NP_175485.1; AtCDPK34: NP_197437.1); Pf: *Plasmodium falciparum* (PfCDPK1: PKC46465.1; PfCDPK2: PKC43076.1; PfCDPK3: PKC45344.1; PfCDPK4: PKC42240.1; PfCDPK5: PKC47007.1; PfCDPK6: PKC49223.1; PfCDPK7: PKC49078.1); Tg: *Toxoplasma gondii* (TgCDPK1: EPT31305.1; TgCDPK2: EPT27057.1; TgCDPK3: EPT27420.1; TgCDPK4: XP_018637922.1; TgCDPK5: EPT26997.1; TgCDPK6: EPT24667.1; TgCDPK7: KFH12789.1; TgCDPK8: KFH12687.1; TgCDPK9: EPT24739.1); Tt: *Tetrahymena thermophile* (TtCDPK1 (kinase domain protein):

XP_001008217.1, TtCDPK2 (kinase domain protein): XP_001025095.2 and TtCDPK3 (S/T kinase domain protein): XP_001026079.2); Tb: *Trypanosoma brucei* (TbCDPK1: Tb927.2.1820 and TbCDPK2: Tb927.10.3900); Li: *Leishmania infantum* (LiCDPK1: LinJ.33.1810 and LiCDPK2: LinJ.35.0480).

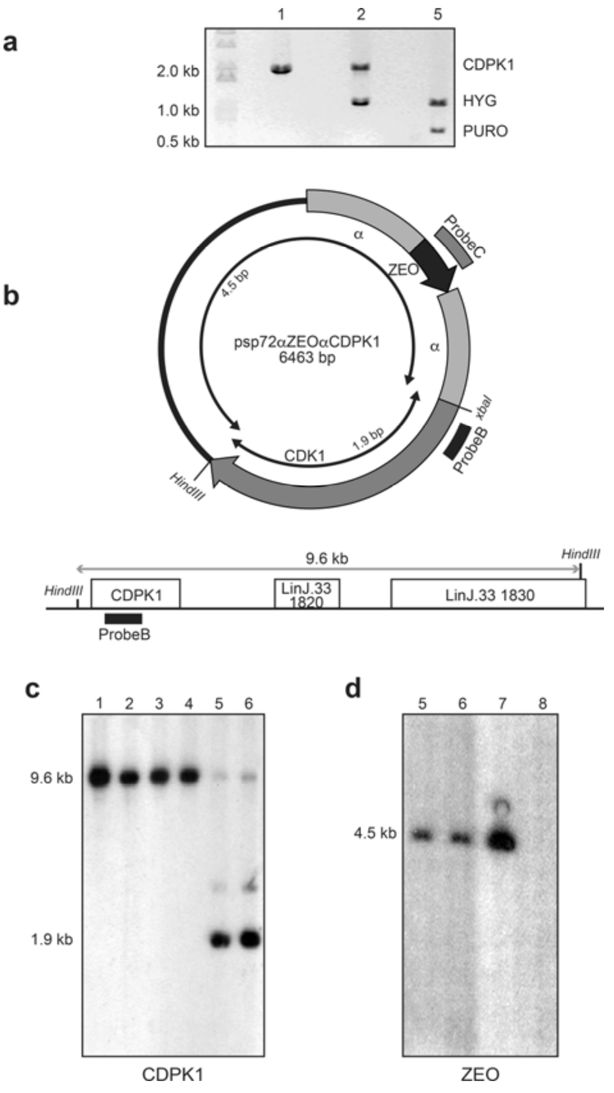

Supplementary Figure 5. **Curing of episomal CDPK1 from CDPK1$^{H/P}$. a,** PCR analyses of DNAs of *Leishmania* lines inactivated for CDPK1. PCR primers are described in Fig. 3a. *L. infantum* wild-type (1); *CDPK1*$^{+/H}$ (2); and *CDPK1*$^{H/P}$+*CDPK1* at passage 3 (5). **b,** Schematic representation of cloned (in pSP72αZEOα) and chromosomal *CDPK1*. **c,** Southern blot analysis with genomic DNAs digested with XbaI and HindIII from the *L. infantum* wild-type (1) and recombinant clones of *CDPK1*$^{+/H}$ (2); *CDPK1*$^{+/P}$ (3); *CDPK1*$^{H/P}$ (4); *CDPK1*$^{H/P}$+*CDPK1* from passage 3 (5) and *CDPK1*$^{H/P}$+*CDPK1* from passage 50 (6). The 9.6 kb fragment corresponds to chromosomal copy of *CDPK1* while the 1.9 kb or 4.5

kb bands are diagnostics of the episome. Hybridization was performed with a probe covering an internal 480 bp region from the *CDPK1* gene (probe B in panel A). **d,** Similar analysis but with genomic DNA derived from *CDPK1*$^{H/P}$+*CDPK1* from passage 3 (5); *CDPK1*$^{H/P}$+*CDPK1* from passage 50 (6); and *CDPK1*$^{+/H}$+*CDPK1* at passage 3 (7) or passage 25 (8). Hybridization was performed with a probe covering a 400 bp region internal to the zeocin resistance marker (probe C). *H* and *P* refer to the *HYG*- and *PURO*-inactivated alleles, respectively. + refers to the wild-type allele. Source data are provided as a Source Data file.

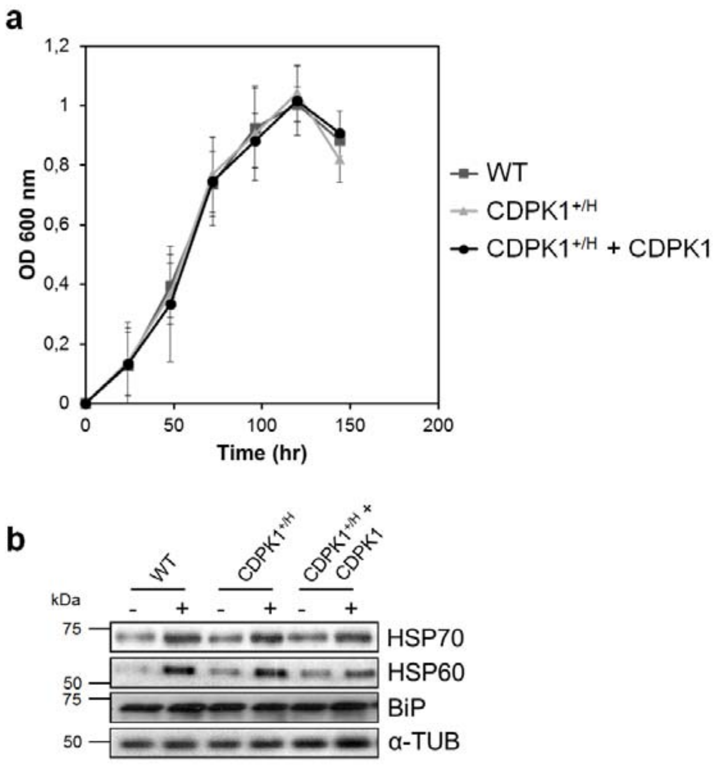

Supplementary Figure 6. **Growth properties and monitoring of stress proteins expression in CDPK1$^{+/H}$ cells. a.** Growth curves of wild-type, *CDPK1*$^{+/H}$ and *CDPK1*$^{+/H}$ + *CDPK1* add-back cells as measured by optical density at 600 nM over time. Data are mean ± SEM for n=3 independent biological replicates. Statistical analyses were performed using unpaired t-tests. **b,** Expression of stress proteins in cells grown at room temperature (-) or heat stress (37°C) (+). The quantity of proteins layered in each lane was monitored with an anti-tubulin antibody (α-TUB). *H* and + refer to the *HYG*-inactivated and wild-type alleles, respectively. Source data are provided as a Source Data file.

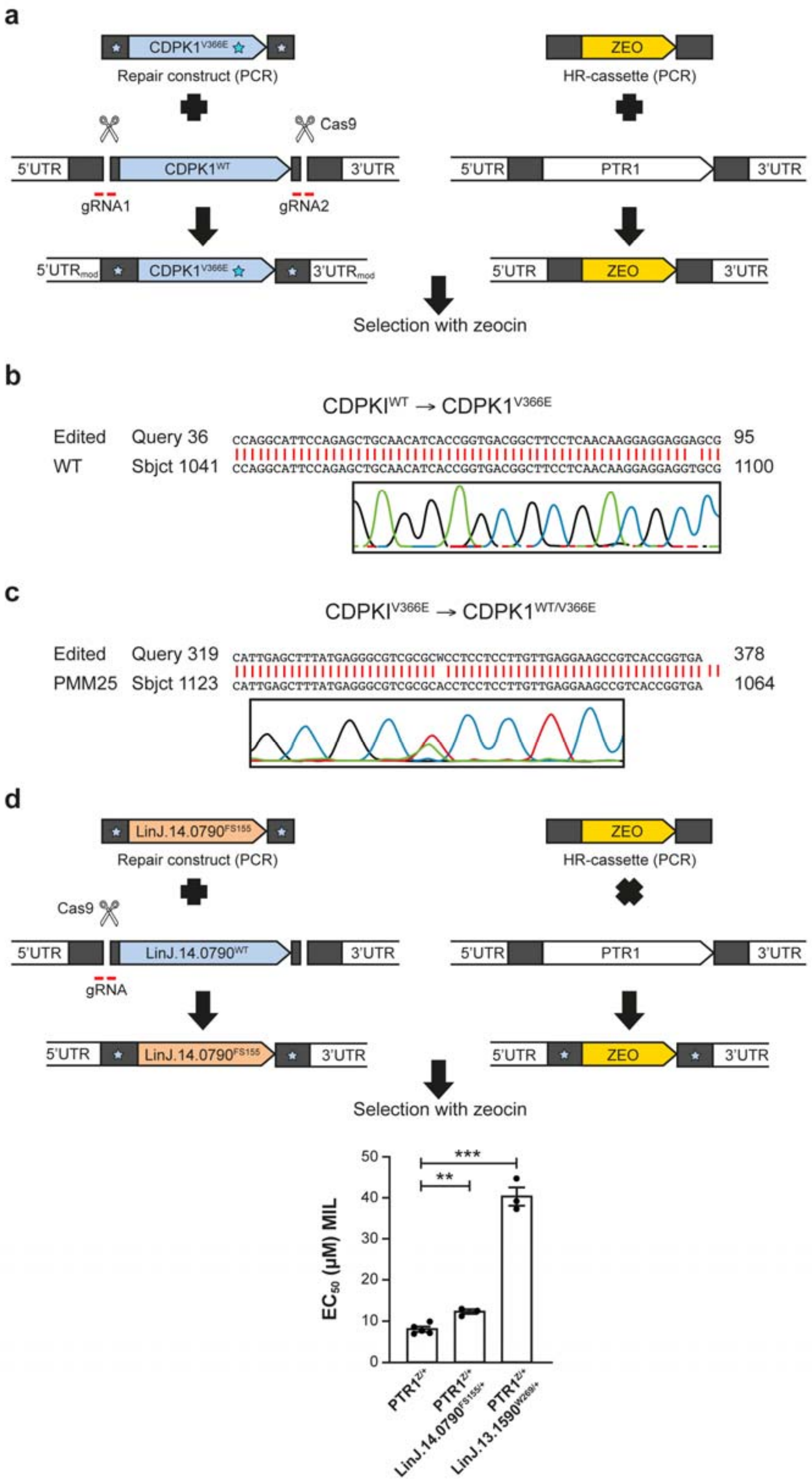

Supplementary Figure 7. **Gene editing using CRISPR-Cas9. a,** A strategy to perform gene editing of *Leishmania CDPK1* was developed using a combination of CRISPR-

Cas9-assisted gene targeting and homologous recombination-based allelic replacement. Two cassettes were prepared. The first cassette is used for repairing the Cas9-targeted site and was amplified using primers specific to the protospacer-adjacent motifs (PAM) for gRNA1 and gRNA2 but in which the PAM site was destroyed. This prevented the repair cassette to be targeted by Cas9. The other cassette was amplified from a construct aiming to knock-out the *PTR1* gene by homologous recombination, using zeocin as a selection marker. The cassettes, along with gRNA-CrRNA hybrids, were co-transfected in *L. infantum* cells expressing Cas9 (*Li*Cas9). The cells were selected using zeocin. Editing was confirmed by PCR amplification of the *CDPK1* gene in zeocin-resistant parasites followed by conventional Sanger sequencing. Chromatogram (inset) and sequence alignment confirmed the integration of the mutant allele in wild-type cells (**b**) and of the wild-type allele in mutant cells (**c**). **d**, We used a similar DNA editing approach for *LinJ.14.0790* and *LinJ.13.1590* involved in miltefosine resistance where a PCR fragment containing a frameshift (LinJ.14.0790 FS155) or a non-sense mutation (LinJ.13.1590 W269*) was transfected in a wild-type cell along with the *ZEO*-containing *PTR1*-targeting construct. Zeocin-resistant parasites had *LinJ.14.0790* or *LinJ.13.1590* alleles edited and this increased resistance to miltefosine. Data are mean ± SEM for n=5 ($PTR1^{Z/+}$) and n= 3 (other samples) independent biological replicates. Statistical analyses were performed using unpaired two-tailed t-tests. *Z* and + refer to the *ZEO*-inactivated and wild-type alleles, respectively. Source data are provided as a Source Data file.

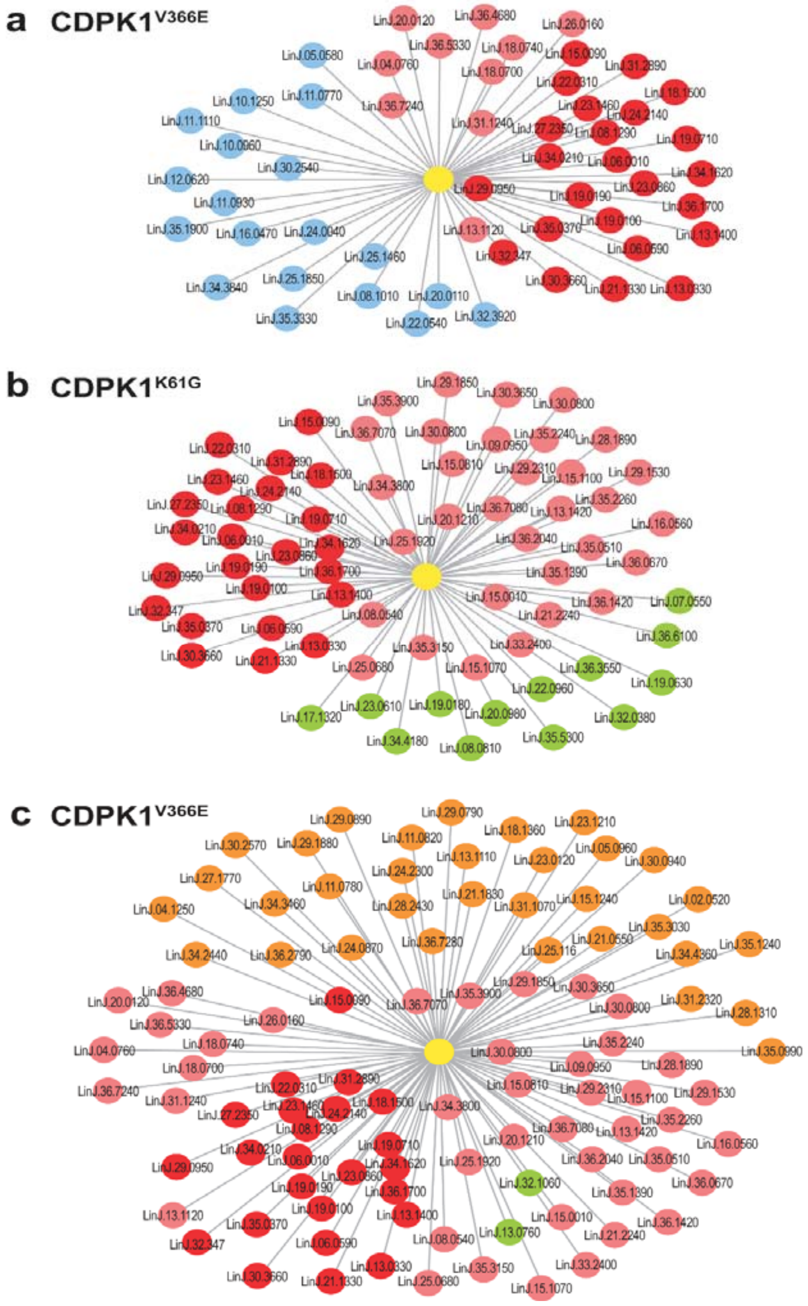

Supplementary Figure 8. **Visualization of predicted interacting partners for CDPK1 versions.** This is an enlarged version of Fig. 5a for facilitating the reading of the gene IDs of the interacting proteins. Nodes inferring specific gene-IDs as retrieved from SAINT analysis (average P >0.8) of IP data for CDPK1-HA, CDPK1^{K61G}-HA and CDPK1^{V366E}-HA. SAINT analysis was performed using total spectral counts for peptides identified with

<1% FDR. Unique nodes are presented in blue, green and orange for CDPK1-HA (**a**), CDPK1^{K61G}-HA (**b**) and CDPK1^{V366E}-HA (**c**), respectively. Common proteins are presented in shades of red depending on the level of overlap.

a

SampleID	Gene	Product	Molecular weight	Rational
1	LinJ.06.0590	60S ribosomal protein L23a (rL23a)	16336	IP all
2	LinJ.11.1110	60S ribosomal protein L28	16370	IP WT
3	LinJ.35.0370	ATP-dependent DEAD-box RNA helicase	46394	IP all
4	LinJ.34.0210	ARM56	56085	IP all; motif
5	LinJ.10.0960	small GTP-binding protein Rab11	23402	IP WT
6	LinJ.10.1250	Ras-related protein RabX1	24548	IP WT; motif
7	LinJ.28.2950	HSP70	71267	Phospho prot.; motif
8	LinJ.34.1620	p25-alpha	16616	IP all

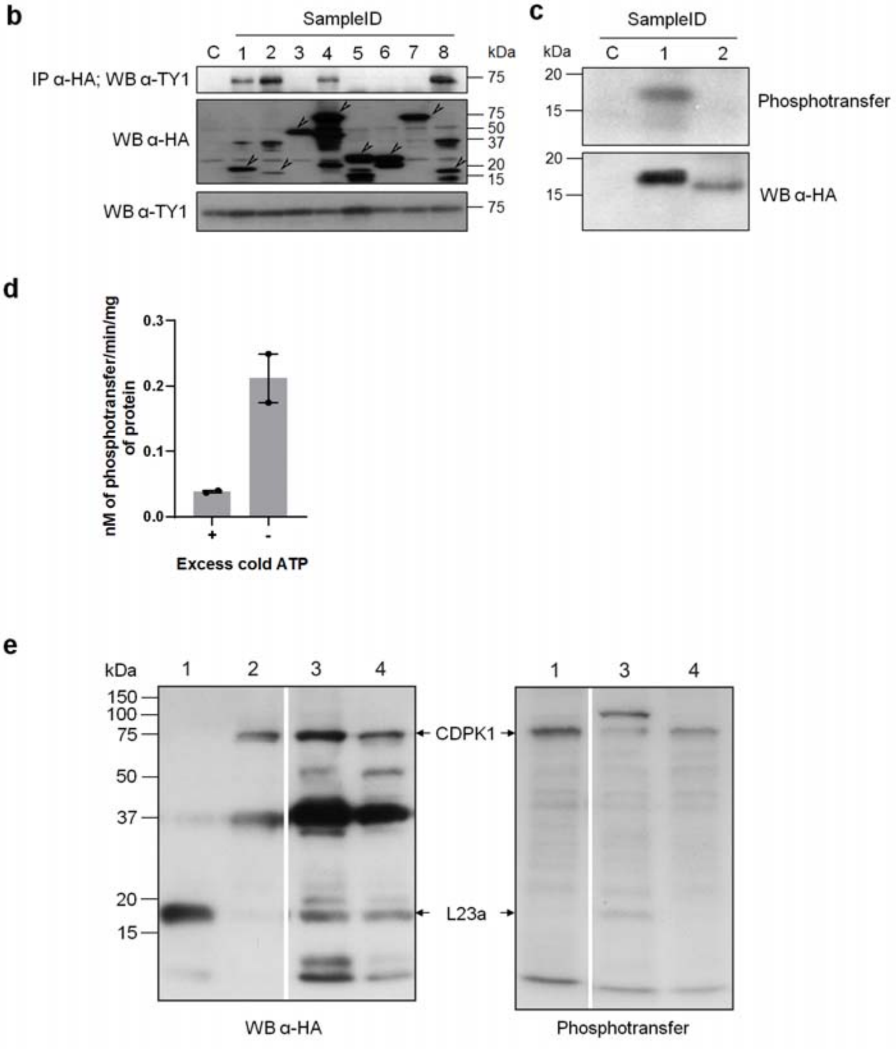

Supplementary Figure 9. **Reciprocal immunoprecipitation of potential partners of CDPK1 and CDPK1 mediated phosphorylation of ribosomal protein L23a. a**, List of

putative CDPK1 partners (prey proteins) assessed by reciprocal immunoprecipitation and rational for their selection. IP all: the protein immunoprecipitated with the three versions of CDPK1 (WT, K61G and V366E in Supplementary Table 5); IP WT: the protein immunoprecipitated only with the wild-type version of CDPK1 (Supplementary Table 5); motif: the protein is predicted to contain an AMPK/CaMK phosphorylation motif; Phospho prot: the protein was previously detected in a phosphoproteome study. **b**, Lysates of cells expressing TY1-CDPK1 and HA-tagged versions of prey proteins (see panel A) were immunoprecipitated with anti-HA antibody followed by western blotting using anti-TY1 antibody for assessing the co-immunoprecipitation of TY1-CDPK1 (upper panel). Expression of HA-tagged versions of the prey proteins (indicated by arrow heads in the middle panel) and of TY1-CDPK1 (lower panel) in each of the lysates was confirmed by western blot using anti-HA and anti-TY1 antibody, respectively. **c,** Immunoprecipitation (using anti-HA) coupled to in vitro kinase assay was performed with *L. infantum* expressing HA-CDPK1 co-transfected with empty vector (C) or a vector expressing HA-LinJ.06.0590 (1) or HA-LinJ.11.1110 (2) (upper panel). Western blot performed with anti-HA antibody on the immunoprecipitates used for the kinase assay confirms the expression of the target proteins (lower panel). **d,** In vitro kinase assay was performed with immunoprepitates from lysates of *CDPK1*$^{+/H}$ cells expressing HA-CDPK1. Assays were performed in presence (+) or absence (-) of excess cold ATP to confirm active phosphotransfer to the AMARA peptide. AMARA peptide is a minimal substrate for several members of the protein kinases family. It contains the phosphorylation site for AMP-activated Protein Kinase and is employed to measure AMPK-related kinase activity. Results are representative of n=2 biologically independent experiments. **e,**

Immunoprecipitation (using anti-HA) coupled to in vitro kinase assay was performed with *L. infantum* expressing HA-LinJ.06.0590 (L23a) (1), HA-CDPK1 (2), HA-LinJ.06.0590 along with HA-CDPK1 (3), or HA-LinJ.06.0590 (L23a) along with HA-CDPK1^{K61G} (4) (left panel). The wild-type version of CDPK1 is required for the phosphorylation of LinJ.06.0590 (L23a) (right panel). The Source data are provided as a Source Data file.

Supplementary Tables

Supplementary Table 1. Colonies growing on plates following mutagens treatment and selection with

MIL and PMM at 5× or 10× their EC_{50}.

Drug[a]	Mutagen[b]	Mutagen conc.[c]	# clones obtained at [drug] (clone IDs)[d]	
			5× EC_{50}	10× EC_{50}
**MIL**	None	None	0	0
	EMS	40 mM	7 (MIL 1-7)	3 (MIL 8-10)
	ENU	4 mM	0	0
	MMS	0.1 mM	0	0
	HPMA	100 mM	6 (MIL 11-16)	0
**PMM**	None	None	0-5[e]	0
	EMS	40 mM	40[e]	11 (PMM 1-11)
	ENU	4 mM	56[e]	11 (PMM 12-22)
	MMS	0.1 mM	42[e]	3 (PMM 23-25)
	HPMA	100 mM	0	0

[a] MIL, miltefosine; PMM, paromomycin.

[b] EMS, Ethyl methanesulphonate; ENU, Ethylnitrosourea; MMS, Methylmethane-sulphonate; HMPA, Hexamethylphosphoramide.

[c] Concentration of mutagen used for chemical mutagenesis of parasites prior to their selection with either MIL or PMM.

[d] The number of clones obtained after plating mutagenized parasites in the presence of MIL or PMM at their 5× EC_{50} or 10× EC_{50} concentrations.

[e] These clones were not analyzed further.

Supplementary Table 2. List of homozygous mutations detected in miltefosine resistant mutants.

Common in	GeneID	Product[a]	Mutant(s)	Pos. in protein	From[b]	To[b]
1	LinJ.12.0662	surface antigen protein 2, putative	MIL8	27	T	K
1	LinJ.13.1370	mitochondrial DFS polymerase I protein D, putative	MIL7	643	G	D
1	LinJ.13.1430	protein Associated with Differentiation, putative	MIL9	77	G	E
1	LinJ.13.1590	miltefosine transporter*	MIL7	269	W	*
1	LinJ.14.1180	kinesin K39, putative	MIL7	2153	E	D
1	LinJ.14.1180	kinesin K39, putative	MIL9	2156	K	R
1	LinJ.14.1180	kinesin K39, putative	MIL9	2158	A	T
1	LinJ.15.1540	cAMP specific phosphodiesterase, putative	MIL8	186	T	I
1	LinJ.17.0360	hypothetical protein, conserved	MIL9	341	A	V
1	LinJ.22.1570	hypothetical protein	MIL7	207	M	I
1	LinJ.33.2140	hypothetical protein, unknown function	MIL9	67	WT	FS
1	LinJ.34.0360	ATPase family associated with various cellular activities (AAA), putative	MIL8	124	R	G
1	LinJ.34.0710	flagellar attachment zone protein, putative	MIL1	240	WT	FS
1	LinJ.35.4610	uncharacterized conserved protein, putative	MIL12	142	WT	FS
1	LinJ.36.0050	PUF1, putative	MIL10	278	H	R
1	LinJ.36.6220	glycerophosphoryl diester phosphodiesterase, putative*	MIL10	46	WT	FS
2	LinJ.14.1180	kinesin K39, putative	MIL7,9	2162	R	Q
2	LinJ.35.0520	proteophosphoglycan ppg4	MIL7, 8	1091	L	V
2	LinJ.35.0550	proteophosphoglycan ppg1	MIL1, 2	203	F	I
3	LinJ.27.0250	kinetoplast-associated protein-like protein (fragment)	MIL1, 4, 7	656	WT	FS
4	LinJ.14.0790	fatty acid elongase, putative*	MIL6, 7, 8, 10	155	WT	FS
4	LinJ.30.1310	pyridoxal kinase,putative	MIL1, 2, 3, 4	197	H	D
5	LinJ.20.0750	hypothetical protein, conserved	MIL6, 7, 9, 10, 15	1042	WT	FS
7	LinJ.12.0670	surface antigen protein 2, putative	MIL1, 5, 6, 7, 9, 10, 12	403	WT	FS
10	LinJ.15.0500	hypothetical protein	MIL1, 2, 3, 4, 5, 6, 7, 8, 9, 10	2058	WT	FS
11	LinJ.20.1200	hypothetical protein	MIL6, 7, 8, 9, 10, 11, 12, 13, 14, 15, 16	581	WT	FS

[a] Genes tested by episomal expression of the wild-type gene in mutants are marked with an asterisk.

[b] Amino acids alterations are indicated for single-nucleotide substitutions. Indels lead to frameshift (FS) mutations.

Supplementary Table 3. List of homozygous mutations detected in the various paromomycin resistant mutants

Common in	GeneID	Product[a]	Mutant(s)	Pos. in protein	From[b]	To[b]
**1**	LinJ.16.1320	hypothetical protein, conserved	PMM3	2187	W	*
**1**	LinJ.11.1260	ATP-binding cassette protein subfamily A, member 5, putative	PMM15	66	I	V
**1**	LinJ.27.1660	tubulin cofactor C domain-containing protein 1, putative *	PMM18	341	L	F
**1**	LinJ.33.1810	protein kinase,putative (CDPK1)*	PMM25	366	V	E
**3**	LinJ.15.0500	hypothetical protein	PMM5, 23, 24	2058	WT	FS
**5**	LinJ.27.0250	kinetoplast-associated protein-like protein (fragment)	PMM3,6, 17, 22, 23,	656	WT	FS
**6**	LinJ.12.0670	surface antigen protein 2, putative	PMM7, 12, 15, 16, 20, 23	403	WT	FS

[a] Genes tested by episomal expression of the wild-type gene in mutants are marked with asterisk.

[b] Amino acids alterations are indicated for single-nucleotide substitutions. Indels lead to frameshift (FS) mutations. Asterisks refer to stop codons.

Supplementary Table 4. Genes with recurrent 5' or 3'UTR mutations among mutants.[a]

	Drug	Mutants[b]	Gene	Mutation[c]	Type[d]	Function	Expression (Mutant/WT)[e]	Fold change susceptibility[f]
**5'UTR**	**MIL**	**MIL6**, MIL7, MIL8, MIL9, MIL10, MIL13, MIL15, MIL16	LinJ.07.0420	ΔG at -73	M/M	homoserine dehydrogenase like protein	0.80 ± 0.27	nd
		MIL1, MIL2, MIL4, MIL5, MIL6, MIL7, MIL8, MIL9, MIL10, MIL12, MIL13, **MIL15**, MIL16	LinJ.14.0340	Insert. C at -303	M/M	inositol polyphosphate kinase like protein	1.67 ± 0.56*	1.1× (n=3)[ns]
		MIL6, MIL7, MIL8, MIL9, MIL11, MIL12, MIL13, **MIL15**	LinJ.36.6160	Insert. T at -465	+/M	choline-ethanolamine phosphotransferase	0.84 ± 0.30	nd
	**PMM**	PMM1, PMM2, PMM3, PMM4, PMM6, PMM7, PMM8, PMM9, PMM10, PMM11, PMM12, PMM13, PMM14, PMM15, PMM16, PMM17, PMM18, PMM19, PMM20, PMM21, PMM22, PMM23, **PMM24**, PMM25	LinJ.10.1100	ΔAC at -314	M/M	carrier protein	0.81 ± 0.26	nd
		PMM1, PMM2, PMM4, PMM8, PMM9, PMM10, PMM11, PMM12, PMM13, PMM15, PMM16, **PMM18**, PMM20, PMM21, PMM22, PMM23, PMM24	LinJ.29.1500	ΔAT at -203	M/M	RNA binding protein, putative	1.04 ± 0.33	nd
		PMM2, PMM7, PMM10, PMM13, PMM16, PMM20, PMM21, **PMM24**	LinJ.35.0790	Insert. GC at -325	+/M	rRNA dimethyltransferase, putative	0.71 ± 0.24*	1.0× (n=3)[ns]
**3'UTR**	**MIL**	**MIL6**, MIL7, MIL8, MIL9, MIL16	LinJ.09.0080	ΔC or ΔCC at +482	M/M	RNA binding protein, putative	0.51 ± 0.12**	nd
		**MIL6**, MIL7, MIL8, MIL9, MIL10, MIL11, MIL12, MIL15, MIL16	LinJ.34.0690	Insert. A at +329	M/M	ABCC8 transporter	0.42 ± 0.15***	1.6× (n=3)***
	**PMM**	PMM1, PMM2, PMM9, PMM10, PMM17, PMM18	LinJ.14.0080	ΔG at +544	M/M	Hypothetical protein	0.66 ± 0.20*	1.0× (n=6)[ns]

	PMM3	PMM11	PMM20
	PMM4	PMM12	PMM21
	PMM5	PMM13	PMM22
	PMM6	PMM14	PMM23
	PMM7	PMM15	**PMM24**
	PMM8	PMM16	PMM25

[a] Mutations were searched within the 650 nucleotides upstream of the ATG of the genes (5'UTR) or the 750 nucleotides downstream of their stop codon (3'UTR). Genes with 5' or 3' UTRs mutations in multiple mutants (not necessarily at the exact same position) were considered as mutated in a recurrent fashion.

[b] The names of the mutants in which mutations were detected in the UTR of the gene are indicated. For each gene, the mutant used for functional characterization is indicated in bold.

[c] The mutation detected in the mutant used for functional characterization (i.e. mutant in bold in column 3). Δ, deletion; Insert., Insertion. For 5'UTRs, the nucleotide positions upstream (-) of the ATG are indicated. For 3'UTRs, the nucleotide positions downstream (+) of the stop codon are indicated.

[d] M/M, homozygous mutation; +/M, heterozygous mutation.

[e] Gene expression was compared between the mutants and the parental wild type (WT) clone by qRT-PCR. The mutant/WT expression ratios are shown. *, $p<0.05$; **, $p<0.01$; *** $p<0.001$.

[f] Genes with a significantly downregulated expression in the mutant were transfected in the mutants and we monitored for drug resensitization among transfectants. Genes with a significantly upregulated expression in the mutant were transfected in wild-type *L. infantum* and we monitored for increased drug resistance among transfectants. Statistical analyses were performed using

unpaired two-tailed t-tests. ***, $p<0.001$; ns, not significant. nd, not done. The increase in gene expression following transfection of wild-type cells with an episome coding for LinJ.14.0340 was 8.48 ± 3.64 fold (*p*-value 0.0116). The increase in gene expression following transfection of mutants with episomes coding for LinJ.14.0080 (in PMM24), LinJ.34.0690 (in MIL6) and LinJ.35.0790 (in PMM24) was 2.55 ± 1.20 fold ($p = 0.0005$), 2.34 ± 1.01 fold ($p = 0.0042$) and 3.33 ± 1.53 fold ($p = 0.0070$), respectively.

Supplementary Table 5. List of proteins from the Venn diagram of Fig 4b. The 24 Proteins detected in immunoprecipitates from all three versions of CDPK1 (WT, K61G and V366E) are indicated on the right, the 11 proteins detected specifically in immunoprecipitates from the WT and V366E versions are indicated in the middle and the 18 proteins found exclusively with the wild-type version of CDPK1 are indicated on the left.

Proteins detected exclusively in immunoprecipitates from the WT CDPK1			Proteins detected in immunoprecipitates from the WT and V366E versions of CDPK1			Proteins detected in immunoprecipitates from the WT, K61G and V366E versions of CDPK1		
Total	GeneID	Product description	Total	GeneID	Product description	Total	GeneID	Product description
18	LinJ.24.0040	60S ribosomal protein L17, putative	11	LinJ.13.1120	40S ribosomal protein S4, putative	24	LinJ.22.0310	40S ribosomal protein S15, putative
	LinJ.35.1900	60S ribosomal protein L36, putative		LinJ.11.0770	40S ribosomal protein S21, putative		LinJ.06.0590	60S ribosomal protein L23a, putative
	LinJ.35.3330	60S ribosomal subunit protein L31, putative		LinJ.26.0160	60S ribosomal protein L7, putative		LinJ.24.2140	60S ribosomal protein L26, putative
	LinJ.16.0470	60S ribosomal protein L21, putative		LinJ.18.0740	Elongation factor Tu, mitochondrial, putative		LinJ.35.0370	ATP-dependent DEAD-box RNA helicase, putative
	LinJ.11.1110	60S ribosomal protein L28, putative		LinJ.04.0760	nascent polypeptide associated complex subunit- like protein, copy 2		LinJ.21.1330	T-complex protein 1, delta subunit, putative
	LinJ.22.0540	prefoldin 5-like protein		LinJ.36.7240	T-complex protein 1, theta subunit, putative		LinJ.23.1460	T-complex protein 1, gamma subunit, putative
	LinJ.30.2540	heat shock 70-related protein 1, mitochondrial precursor, putative		LinJ.18.0700	citrate synthase, putative		LinJ.13.1400	chaperonin TCP20, putative
	LinJ.10.1250	Ras-related protein RabX1, putative		LinJ.20.0120	phosphoglycerate kinase B, cytosolic		LinJ.27.2350	heat shock protein DNAJ, putative
	LinJ.10.0960	small GTP-binding protein Rab11, putative		LinJ.31.1240	Pyrophosphate-energized vacuolar membrane proton pump 1, putative		LinJ.32.3470	chaperonin alpha subunit, putative
	LinJ.25.1460	GTP-binding protein, putative		LinJ.36.4680	hypothetical protein, conserved LinJ.36.4680		LinJ.15.0090	ATP-dependent protease ATPase subunit HslU1, putative
	LinJ.32.3920	kinetoplast-associated protein p18-2, putative		LinJ.36.5330	hypothetical protein, conserved LinJ.36.5330		LinJ.31.2890	ADP-ribosylation factor, putative
	LinJ.12.0620	cytochrome oxidase subunit IV, putative					LinJ.29.0950	ADP-ribosylation factor-like protein 3A, putative
	LinJ.25.1850	3-oxo-5-alpha-steroid 4-dehydrogenase, putative					LinJ.36.1700	clathrin heavy chain, putative
	LinJ.20.0110	phosphoglycerate kinase C, glycosomal					LinJ.34.1620	p25-alpha, putative
	LinJ.05.0580	hypothetical protein, conserved LinJ.05.0580					LinJ.18.1500	P-type H+-ATPase, putative
	LinJ.08.1010	hypothetical protein, conserved LinJ.08.1010					LinJ.30.3660	ATP synthase, epsilon chain, putative
	LinJ.11.0930	hypothetical protein, conserved LinJ.11.0930					LinJ.19.0190	ADP/ATP translocase 1, putative
	LinJ.34.3840	hypothetical protein, conserved LinJ.34.3840					LinJ.06.0010	histone H4
							LinJ.19.0710	glycosomal malate dehydrogenase
							LinJ.23.0860	3-ketoacyl-CoA thiolase, putative
							LinJ.34.0210	Antimony resistance marker of 56 kDa
							LinJ.13.0330	alpha tubulin
							LinJ.08.1290	beta tubulin
							LinJ.19.0100	hypothetical protein, conserved LinJ.19.0100

Supplementary Table 6. Predicted targets for CDPK1. 206 proteins retrieved from kinase assay-gel and detected by LC-MS/MS were analyzed for AMPK/CaMK phosphorylation motifs by KinasePhos2.0. Proteins predicted with high confidence (SVMscore > 0.8) are shown. Proteins previously reported to be phosphorylated are highlighted in bold. Proteins that have been identified by CDPK1 IP (see Supplementary Table 5) are indicated by an asterisk.

Gene ID	Product Description
**LinJ.15.1010**	**40S ribosomal protein S3, putative**
LinJ.32.3320	ribosomal protein L3, putative
**LinJ.18.1350**	**heat shock protein 110, putative**
**LinJ.26.1220**	**heat shock protein 70-related protein**
**LinJ.28.2960**	**heat-shock protein hsp70, putative**
LinJ.26.0630	protein disulfide isomerase, putative
LinJ.24.2220	ubiquitin-conjugating enzyme E2, putative
LinJ.10.1250	Ras-related protein RabX1, putative*
LinJ.19.0190	ADP/ATP translocase 1, putative*
LinJ.19.0970	4-coumarate:coa ligase-like protein
LinJ.23.0120	GDP-mannose pyrophosphorylase
LinJ.24.2200	3-hydroxy-3-methylglutaryl-CoA synthase, putative
LinJ.30.0120	alkyldihydroxyacetonephosphate synthase
LinJ.36.4100	S-adenosylhomocysteine hydrolase
LinJ.34.0210	Antimony resistance marker of 56 kDa*
**LinJ.11.0930**	**hypothetical protein, conserved***
