# Supplementary material for: Coupling chemical mutagenesis to next generation sequencing for the identification of drug resistance mutations in Leishmania
Source: Nat Commun. 2019 Dec 9;10:5627. doi: 10.1038/s41467-019-13344-6 (PMC6901541; doi:10.1038/s41467-019-13344-6)
Supplement: Supplementary file 3 — Reporting Summary [file 41467_2019_13344_MOESM3_ESM.pdf]

nature research

Corresponding author(s): Marc Ouellette - NCOMMS-18-29221B

Reporting Summary

Nature Research wishes to improve the reproducibility of the work that we publish. This form provides structure for consistency and transparency in reporting. For further information on Nature Research policies, see Authors & Referees and the Editorial Policy Checklist.

Statistical parameters

When statistical analyses are reported, confirm that the following items are present in the relevant location (e.g. figure legend, table legend, main text, or Methods section).

n/a	Confirmed	
☐	☒	The exact sample size (*n*) for each experimental group/condition, given as a discrete number and unit of measurement
☐	☒	An indication of whether measurements were taken from distinct samples or whether the same sample was measured repeatedly
☐	☒	The statistical test(s) used AND whether they are one- or two-sided<br>*Only common tests should be described solely by name; describe more complex techniques in the Methods section.*
☐	☒	A description of all covariates tested
☐	☒	A description of any assumptions or corrections, such as tests of normality and adjustment for multiple comparisons
☐	☒	A full description of the statistics including central tendency (e.g. means) or other basic estimates (e.g. regression coefficient) AND variation (e.g. standard deviation) or associated estimates of uncertainty (e.g. confidence intervals)
☐	☒	For null hypothesis testing, the test statistic (e.g. *F*, *t*, *r*) with confidence intervals, effect sizes, degrees of freedom and *P* value noted<br>*Give P values as exact values whenever suitable.*
☒	☐	For Bayesian analysis, information on the choice of priors and Markov chain Monte Carlo settings
☒	☐	For hierarchical and complex designs, identification of the appropriate level for tests and full reporting of outcomes
☒	☐	Estimates of effect sizes (e.g. Cohen's *d*, Pearson's *r*), indicating how they were calculated
☐	☒	Clearly defined error bars<br>*State explicitly what error bars represent (e.g. SD, SE, CI)*

Our web collection on statistics for biologists may be useful.

Software and code

Policy information about availability of computer code

Data collection	not applicable
Data analysis	bwa-mem v0.7.5<br>Picard v1.115<br>GATK v3.2.2<br>SAINT v3.6.1<br>Protein Pilot v4.5<br>Mascot v2.5.1<br>Scaffold v4.7.1<br>MEGA6<br>GraphPad Prism v5.01

For manuscripts utilizing custom algorithms or software that are central to the research but not yet described in published literature, software must be made available to editors/reviewers upon request. We strongly encourage code deposition in a community repository (e.g. GitHub). See the Nature Research guidelines for submitting code & software for further information.

April 2018

Data

Policy information about availability of data

All manuscripts must include a data availability statement. This statement should provide the following information, where applicable:

- Accession codes, unique identifiers, or web links for publicly available datasets
- A list of figures that have associated raw data
- A description of any restrictions on data availability

The dataset supporting the conclusions of this article is available in the Sequencing Read Archive (https://www.ncbi.nlm.nih.gov/sra) repository with accession numbers indicated in the Data availability section of the manuscript.

Field-specific reporting

Please select the best fit for your research. If you are not sure, read the appropriate sections before making your selection.

☒ Life sciences ☐ Behavioural & social sciences ☐ Ecological, evolutionary & environmental sciences

For a reference copy of the document with all sections, see nature.com/authors/policies/ReportingSummary-flat.pdf

Life sciences study design

All studies must disclose on these points even when the disclosure is negative.

Sample size	With our experience of deep sequencing in Leishmania, for step wise selection, we use five individual clones for identifying genomic variations pertaining to drug resistance. Considering the randomness of mutagenesis in mut-seq, we decided to use a greater number of clones. For validation with miltefosine we used 16 clones obtained from one round of mutagenesis and 11 out of 16 clones were mutated for the hallmark miltefosine transporter gene. It was thus decided that for paromomycin the genome sequence of 25 clones obtained from the highest drug selection should be sufficient to identify genomic events implicated in resistance.
Data exclusions	No data were excluded.
Replication	Experiments were replicated at least thrice except the polysome profile and kinase competition experiments which were done with two independent replicates.
Randomization	Randomization is not applicable to our study. The genome of all mutants had to be compared to the same wildtype parent clone from which they were derived.
Blinding	Blinding was not applicable to our study for the same reason as explained above.

Reporting for specific materials, systems and methods

Materials & experimental systems

n/a	Involved in the study
☒	☐ Unique biological materials
☐	☒ Antibodies
☐	☒ Eukaryotic cell lines
☒	☐ Palaeontology
☒	☐ Animals and other organisms
☒	☐ Human research participants

Methods

n/a	Involved in the study
☒	☐ ChIP-seq
☒	☐ Flow cytometry
☒	☐ MRI-based neuroimaging

Antibodies

Antibodies used	1. mouse anti-HA monoclonal Antibody, SantaCruz biotechnology, Cat: Sc-7392<br>2. Goat antimouse HRP conjugated antibody, Thermo Scientific, Cat: 31430<br>3. mouse anti-tubulin, Millipore Sigma, Cat : T6199<br>4. mouse anti-HSP70, Abcam Cat: ab2787<br>5. mouse anti-HSP60, Assay Design, Cat: SPA-807-488<br>6. Goat anti-rabbit HRP-conjugated antibody, Abcam, Cat: ab6721<br>7. Rabbit anti-Ty1, GenScript, Cat: A01004
Validation	The validation statement of the manufacturer was considered.

Eukaryotic cell lines

Policy information about cell lines

Cell line source(s)	ATCC
Authentication	None of the cell lines used were authenticated
Mycoplasma contamination	The cell lines were not tested for Mycoplasma contamination
Commonly misidentified lines (See ICLAC register)	Not applicable
