# Supplementary material for: Coupling chemical mutagenesis to next generation sequencing for the identification of drug resistance mutations in Leishmania
Source: Nat Commun. 2019 Dec 9;10:5627. doi: 10.1038/s41467-019-13344-6 (PMC6901541; doi:10.1038/s41467-019-13344-6)
Supplement: Supplementary file 4 — Description of Additional Supplementary Files [file 41467_2019_13344_MOESM4_ESM.pdf]

Description of Additional Supplementary Files

File Name: Supplementary Data 1
Description: contains the list of genes for which we detected single nucleotide variants in at least two of our miltefosine-resistant mutants.

File Name: Supplementary Data 2
Description: contains the list of genes for which we detected single nucleotide variants in at least two of our paromomycin-resistant mutants.

File Name: Supplementary Data 3
Description: contains data pertaining to the interactome of the CDPK1 protein kinase and its variants.

File Name: Supplementary Data 4
Description: contains data pertaining to the identification of proteins from the kinase assay performed with immunoprecipitated CDPK1.

File Name: Supplementary Data 5
Description: is listing the PCR primers used in this study.
